# Altering Antigen Charge to Control Self-Assembly and Processing of Immune Signals During Cancer Vaccination

**DOI:** 10.3389/fimmu.2020.613830

**Published:** 2021-01-06

**Authors:** Shannon J. Tsai, Allie Amerman, Christopher M. Jewell

**Affiliations:** ^1^ Fischell Department of Bioengineering, University of Maryland, College Park, MD, United States; ^2^ Robert E. Fischell Institute for Biomedical Devices, University of Maryland, College Park, MD, United States; ^3^ United States Department of Veterans Affairs, VA Maryland Health Care System, Baltimore, MD, United States; ^4^ Department of Microbiology and Immunology, University of Maryland Medical School, Baltimore, MD, United States; ^5^ Marlene and Stewart Greenebaum Cancer Center, Baltimore, MD, United States

**Keywords:** immunotherapy, vaccine, cancer, immune, polyplex, nanoparticle, co-delivery, self-assembly

## Abstract

Biomaterial delivery systems offer unique potential to improve cancer vaccines by offering targeted delivery and modularity to address disease heterogeneity. Here, we develop a simple platform using a conserved human melanoma peptide antigen (Trp2) modified with cationic arginine residues that condenses an anionic toll-like receptor agonist (TLRa), CpG, into polyplex-like nanoparticles. We reasoned that these structures could offer several useful features for immunotherapy – such as tunable loading, co-delivery of immune cues, and cargo protection – while eliminating the need for synthetic polymers or other complicating delivery systems. We demonstrate that Trp2/CpG polyplexes can readily form over a range of Trp2:CpG ratios and improve antigen uptake by primary antigen presenting cells. We show antigen loading can be tuned by interchanging Trp2 peptides with defined charges and numbers of arginine residues. Notably, these polyplexes with greater antigen loading enhance the functionality of Trp-2 specific T cells and in a mouse melanoma model, decrease tumor burden and improve survival. This work highlights opportunities to control the biophysical properties of nanostructured materials built from immune signals to enhance immunotherapy, without the added complexity or background immune effects often associated with synthetic carriers.

## Introduction

Cancer vaccines present an exciting new strategy to harness the selective ability of the immune system to target tumor cells (1–3). Tumors evade normal immune function in part because they are self-derived cells, minimizing immunogenicity and the warning signals pathogens typically display (4). These innate immune signals are needed to activate dendritic cells (DCs) and other antigen presenting cells to support adaptive immune responses that can combat pathogen or tumors. Additionally, the tumor microenvironment is highly suppressive, hindering the ability of immune cells to maintain anti-tumor responses (5, 6). Improved understanding of the innate immune system has allowed for the discovery and design of new immunomodulatory molecules that can target specific pathways to generate robust pro-immune responses and enhance tumor-specific T cell expansion (7, 8). Many candidate immunotherapeutic approaches are thus exploring agonists that target toll-like receptors (TLR), a family of pathways that recognize pathogen-associated molecular patterns that are expressed on bacteria, viruses, and peptides, or released from stressed or dying cells (9–11). These immunostimulatory molecules can induce potent T cell responses. Along these lines, several FDA-approved immunotherapies have emerged, such as Cervarix (cervical cancer) and Aldara (basal cell carcinoma) that trigger TLR4 and TLR7/8 (12). A number of candidate cancer vaccines are also exploring CpG – a DNA motif commonly found in bacteria – to promote an immunostimulatory cascade and potentiate an antigen-specific immune responses through TLR9 binding and activation (13–15).

Following immunization, DCs take up and present antigen, express co-stimulatory molecules, and produce distinct cytokines that activate and expand cytotoxic T cells (CTLs) able to attack tumors (16). Due to this requirement for dual-presentation of antigen and costimulatory markers, codelivery of antigens and adjuvants can significantly improve the effectiveness of antigen-specific immunotherapies. Thus, the mechanisms of delivery and route of administration remain important considerations (17, 18). As one example, adjuvants delivered alone do not generate durable responses, lack specificity, and can lead to off-target effects. Conversely, delivery of antigens in the absence of immunostimulatory signals can promote immune tolerance (19, 20).

Owing to the potential benefits of precision co-delivery, new molecular-scale and nano-scale delivery systems are being explored to improve immunotherapies (21–23). Nanoparticles as carriers for cancer vaccines, for example, offer a platform for enhancing immune responses through controlled release and targeting to sites such as lymph nodes − tissues that coordinate adaptive immunity. Co-adsorption or co-encapsulation of antigen and adjuvant particles onto polymer scaffolds or inorganic templates also provide co-delivery and control over the internalization of immune signals to generate more potent responses (24, 25). Despite these advantages, many challenges remain to fully realize the benefits of these system, including antigen loading efficiency, more complex manufacturing and regulatory characterization, and heterogenous formulations that can impact safety profiles.

Nanoparticles termed polyplexes have been studied for decades as simple carriers to condense or encapsulate biologic cargo using electrostatic interactions (26–28). Most prominently, synthetic cationic polymers have been developed as gene or protein delivery agents to condense anionic nucleic acid cargo into particulate form that are more readily endocytosed (29). Additionally, by altering polymer structure and function, molecules features can be installed to address barriers to intracellular delivery, such as endosomal escape. While useful, in the vaccine and immunotherapy fields, there are some unique considerations. For example, many polymeric carriers intrinsically activate (30), suppress (31), or alter immune signaling (32) even in the absence of other antigens or adjuvants. These intrinsic immune characteristics can be useful, but can also hinder rational vaccine design and translation because the carrier itself may change the immune response to the antigen or other vaccine components. Additionally, as mentioned above, high-density co-display of antigen and adjuvant is important to generate strong, specific immune responses; in this context polyplexes are particularly well-suited since they by definition juxtapose the condensed components comprising the polyplexes.

To address the issues just highlighted, we assembled polyplex-like structures comprised entirely of immune signals: tumor antigens and TLR ligand. In particular, we assembled CpG – an anionic TLR9 agonist, and a conserved human melanoma peptide (Trp2) modified with arginine residues to create a net cationic charge. This approach of building polyplexes from tumor immunotherapy components offers several attractive design features. First, in contrast to traditional polyplexes, these nanostructures are assembled entirely from immune signals; this unique approach simplifies the design by eliminating the complicating immunogenic effects often associated with carriers or excipients. Secondly, the lack of carriers ensures a high density of immune signals, as 100% of the formulation is cargo. Third, polyplexes maintain many of the attractive features of biomaterial carriers, including a particulate nature for improved uptake, cargo protection, and co-delivery of immune signals. As mentioned, the particulate nature of polyplexes can promote uptake and delivery to internal compartments within cells; these are features that can both be leveraged in immunotherapy design. For example, efficient internalization is critical for antigen processing and subsequent presentation by DCs. Further, many TLRs are located intracellularly within endosomes – including TLR9; agonists such as CpG must therefore be internalized by immune cells to be effective. Uniquely, in our approach, we demonstrate that anionic CpG and cationic Trp2 peptide electrostatically self-assemble to juxtapose antigen and adjuvant for co-delivery without need of synthetic polymers or other carriers. Using this platform, we show that Trp2/CpG polyplexes form over a range of Trp2:CpG ratios and improve antigen uptake by DCs. Treatment of primary DCs strongly activated these cells and promoted Trp2-specific T cell proliferation. Interestingly, polyplexes with higher Trp2:CpG ratios elicit increased inflammatory cytokine production. Leveraging the modularity of this platform, we demonstrate a role of antigen dose using Trp2 peptide modified with different numbers of arginine groups (i.e. 3, 6, and 9). Polyplexes with greater antigen loading enhance T cell functionality which correlates with reduced tumor burden and improved survival in a pre-clinical model of melanoma.

## Materials and Methods

### Synthesis of Trp2:CpG Polyplexes

Trp2/CpG polyplexes were assembled by electrostatic condensation by mixing aqueous solutions of CpG DNA (5’-TCC ATG ACG TTC CTG ACG TT-3’, IDT) and Trp2 peptide (SVYDFFVWL, Genscript) modified with 3(Trp2R_3_), 6 (Trp2R_6_) or 9 (Trp2R_9_) arginine groups. CpG and Trp2R were combined at defined mass ratios ranging from 1:5 to 10:1 Trp2R_x_ : CpG (x=3, 6, 9) by fixing the CpG concentration at 10 µg/mL and varying the amount of Trp2R_x_ under a fixed total volume. Thus, the amount of CpG in each polyplex formulation remained constant irrespective of mass ratio.

### Characterization of Trp2/CpG Polyplexes

Polyplex formation was assessed by SYBR green exclusion assay. 10 µL SYBR Green I at 100X was added to 90 µL reaction mixture of complexes and incubated for 1h. Fluorescence was measured using an excitation wavelength of 497 nm and an emission wavelength of 520 nm. The resulting fluorescence was compared to the average fluorescence of free soluble CpG to determine the fraction of CpG that remained uncondensed. Formation and stability of polyplexes was evaluated by gel retardation assays. 10 µL (100 ng CpG) aliquots of polyplexes were loaded onto a 4% agarose gel with gel loading dye and SYBR Green I (Invitrogen). Electrophoresis was performed at 120V for 20 min in 1X Tris-Borate-EDTA (TBE) buffer. The gel was subsequently imaged using a UV illuminator. The hydrodynamic diameter and zeta potential of complexes were measured in triplicate using a Zetasizer Nano Z590.

### Enzymatic Degradation Assay

CpG was labelled with Cy5 per manufacturer instructions (Mirus, Madison, WI). Polyplexes of varying Trp2R_9_:CpG ratios were made using Cy5-CpG and Trp2R_9_ in 100 uL 1X DNA I reaction buffer. Fluorescence was measured using an excitation wavelength of 640 nm and an emission wavelength of 670 nm to determine initial CpG levels. Fluorescent measurement was chosen to quantify CpG amount over spectrophotometry because peptides (i.e. Trp2R_9_) exhibit absorbance overlap at 260 nm. The complexes were then incubated with 2 units of DNAse I (New England Biolabs) for 30 min at 37°C. Following incubation, the fluorescence was immediately measured again and the extent of degradation of Cy5-CpG in polyplexes was determined by comparing fluorescence intensities relative before and after the addition of DNAse I.

### DC Uptake and Activation

Splenic CD11c^+^ cells were isolated from 6-8 week old female C57BL/6J mice (Jackson Laboratories) through positive selection by magnetic isolation in accordance with manufacturer protocols (Miltenyi Biotech, Cambridge, MA). Cells were plated at 5 x 10^4^ cells per well in 96-well plates with RPMI 1640 media (Lonza, Allendale, NJ) supplemented with 10% FBS, 2mM, L-glutamine, 1X non-essential amino acids, 10mM HEPES buffer (Fisher Scientific, Hampton, NH), 1% penicillin and streptomycin (Gibco), and 55 uM β-mercaptoethanol (Sigma-Aldrich). Cells were treated with either vehicle (water), 200ng of CpG complexed at increasing w/w ratio with Trp2R_9_, or dose-matched soluble CpG and Trp2R_9._ For uptake studies, CpG was labelled with Cy5 per manufacturer instructions (Mirus, Madison, WI) and Cy5 CpG was used to form complexes with FITC-Trp2R_9_ (Genscript). DCs were incubated with treatments for 24h at 37°C, then washed and stained for DAPI, and analyzed by flow cytometry (BD Biosciences). Uptake was also confirmed by confocal microscopy. Splenic CD11c+ cells were isolated and 10^6^ cells were plated onto glass-bottom dishes with No. 1.5 thickness (MatTek). Cells were incubated for 24 h, after which cells were fixed in 4% paraformaldehyde, stained for membrane (wheat germ agglutinin (WGA), Texas Red conjugate) and resuspended in Hoescht for imaging. Images were taken using a Leica SP5X Laser Scanning Confocal and analyzed by FIJI/ImageJ (National Health Institute). For activation studies, DCs were incubated with treatments for 24h, then washed and stained for DAPI and surface activation markers: CD40, CD80, CD86 (BD, San Jose, CA). Cells were then analyzed by flow cytometry.

### DC/T Cell Co-culture

Splenic primary CD11c^+^ cells were isolated as described above, plated at 5 x 10^4^ cells, and treated with polyplexes. After incubation for 24 h, CD8^+^ T cells were isolated from the spleens of Trp2-clone 37 mice (a gift from Dr. Giorgio Trinchieri, National Cancer Institute, NIH), a Trp2-specific transgenic strain, using negative selection *via* magnetic isolation in accordance with manufacturer instructions (StemCell Technologies, Vancouver, BC). Trp2-specific T cells were then labelled by incubating with 50 µM 5(6)Carboxy-fluorescein diacetate N-succinimidyl ester (CFSE) (Sigma Aldrich) per mL of cells for 5 min at room temperature, followed by washing in media. 1.5 x 10^5^ of CFSE labelled Trp2 T cells were then added to treated DC and cultured for an additional 72 h. Following incubation, cells were collected and stained for T cell specific surface markers (CD3e, CD8a), and resuspended in DAPI. T cell proliferation was determined by flow cytometry to measure CFSE dilution.

### Enzyme-Linked Immunosorbent Assay (ELISA)

Supernatants from DC/T cell cultures were collected. Cytokine secretion levels were analyzed *via* ELISA for mouse interferon gamma (IFN-γ) secretion, following manufacturer instructions (BD). 96-well plates were coated with an IFN-γ capture antibody and incubated overnight. Supernatant samples were then added and allowed to bind for 2 hr, followed by an IFN-γ detection antibody and streptavidin-horseradish peroxidase conjugate mixture for 1 h. A tetramethylbenzidine and hydrogen peroxide mixture was added to each well; this reaction was stopped by the addition of 1 M phosphoric acid. Absorbance was read at 450 nm and IFN-γ concentrations were calculated from absorbance by comparing to a standard curve.

### Murine Tumor Models

All studies involving mice (as a source of primary cells) were carried out in compliance with federal, state, and local laws and followed institutional guidelines, including the Guide for the Care and Use of Laboratory Animals and the Animal Welfare Act. All experiments were reviewed and approved by the University of Maryland’s Institutional Animal Care and Use Committee (IACUC). Mice were shaved and injected subcutaneously (s.c.) with 3x10^5^ B16-F10 cells in 50 µL of PBS, and weighed and monitored daily for tumor growth. Tumor size was determined as the product of two orthogonal diameters. Mice were treated s.c. with 50 µL of PBS or 25 µg Trp2R_x_/CpG polyplex treatments when aggregate tumor burden reached 25 mm^2^. Each group received additional doses every 3 days for up to four total treatments. Mice were euthanized according to the IACUC-approved humane endpoints when aggregate tumor burden reached 150 mm^2^.

### Statistics

One-way ANOVA was used to compare three or more groups, with Tukey post-test corrections for multiple comparisons. Log-rank tests were used in analyses of survival. All tests were two-sided analyses and were performed using GraphPad Prism. Error bars in all panels represent mean ± standard error of the mean and p values < 0.05 were considered significant.

## Results

### Trp2R_9_ and CpG Self-Assemble Into Polyplex Nanoparticles

**Figure 1:**
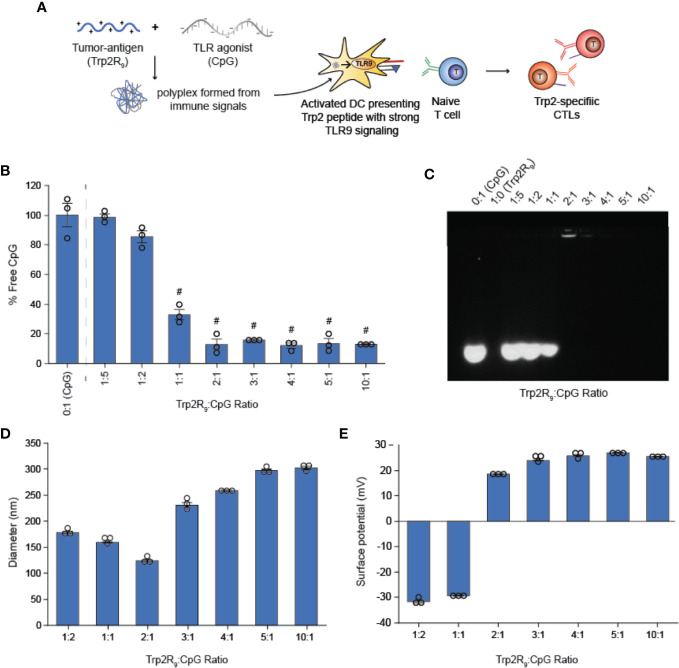
Trp2R_9_ and CpG self-assemble to form polyplexes **(A)** Schematic of polyplex assembly to activate cytotoxic T cells (CTLs) **(B)** SYBR Green exclusion assay confirms formation of polyplexes form at 1:1 Trp2R_9_:CpG ratio **(C)** Gel retardation assay demonstrating the stability of polyplexes above 2:1 Trp2R_9_:CpG ratio **(D)** Hydrodynamic diameter measurements of polyplexes confirming formation of polyplex nanoparticles. **(E)** Surface charge is readily tunable by altering the amount of Trp2R_9_ during polyplex assembly. ^#^p < 0.0001. Statistical comparisons are vs. 0:1 (CpG).

We first tested if polyplexes could be electrostatically assembled from CpG and Trp2 modified with cationic arginine residues (Trp2R_9_) (**Figure 1A**). In these studies, the mass of CpG was fixed while the mass of Trp2R_9_ was varied over a range of 1:5-10:1 Trp2R_9_:CpG (w/w). This weight ratio range corresponds to a cationic:anionic molecular charge ratio of 0.99-4.94 (**Table 1**). To determine the ratios at which polyplex formation occurred, we performed a SYBR green exclusion assay. SYBR green II binds secondary and tertiary structural features on single-stranded DNA by intercalating local regions of stacked base pairs. While unbound SYBR green displays low levels of fluorescence, when bound to DNA, the dye undergoes structural changes resulting in strong fluorescence that increases with nucleic acid concentration. In these studies, the fluorescent intensity of SYBR Green II was measured by UV-vis spectroscopy and normalized to soluble CpG to determine the amount of free CpG remaining. Soluble CpG controls incubated with SYBR Green II led to a high level of fluorescence **(** **Figure 1B** **)**. We observed a significant reduction in fluorescence at 1:1 ratio of Trp2R_9_:CpG and higher ratios, indicating condensation of CpG into polyplexes. To evaluate the stability of polyplexes, we performed gel migration assays. Electrophoresis of polyplexes loaded in agarose gels confirmed that polyplexes do not form below 1:1 Trp2R_9_:CpG ratios (**Figure 1C** **)**. This was indicated by the presence of free CpG under applied voltage. Disappearance of fluorescence above 1:1 Trp2R_9_:CpG ratio indicated quenching of tightly bound nucleic acids and formation of larger complexes that did not migrate. Our studies revealed weak binding of Trp2R_9_ and CpG at a 1:1 Trp2R_9_:CpG weight ratio, as indicated by the presence of a CpG band under applied voltage. Disappearance of fluorescence at 2:1 or higher ratios – even though CpG was present at the same concentration in all wells – confirmed that CpG was fully condensed and Trp2R_9_ and CpG formed stable polyplexes. These findings corresponded with the reduced fluorescence measured in the quantitative exclusion assays.

**Table 1:** Physicochemical Properties of Trp2R_x_/CpG polyplexes.

Trp2R_x_ : CpG (w/w)	Trp2R_x_ : CpGCharge Ratio			Diameter (nm)			Surface Charge (mV)			Composition (% Trp2)			Composition (% CpG)		
	x=3	x=6	x=9	x=3	x=6	x=9	x=3	x=6	x=9	x=3	x=6	x=9	x=3	x=6	x=9
**1:1**	0.39	0.76	0.99	*	145.83 ± 6.87	134.60 ± 2.12	*	-24.57 ± 0.21	-34.43 ± 0.69	64.6	60.1	55.0	35.4	39.9	45.0
**2:1**	0.78	1.51	1.98	81.34 ± 8.88	268.47 ± 19.23	120.77 ± 1.80	-26.10 ± 1.31	18.87 ± 0.50	-25.03 ± 0.41	72.5	59.2	59.0	27.5	40.8	41.0
**3:1**	1.17	2.26	2.96	199.7 ± 17.26	816.40 ± 60.73	128.63 ± 1.40	-11.53 ± .067	22.13 ± 0.21	21.63 ± 0.17	75.5	61.0	64.7	24.5	39.0	35.3
**4:1**	1.55	3.02	3.95	1765.6 ± 83.28	882.40 ± 37.08	159.87 ± 2.01	-.57 ± .13	24.73 ± 0.32	26.60 ± 0.35	75.3	65.1	66.1	24.7	34.9	33.9
**5:1**	1.94	3.77	4.94	1664.3 ± 160.73	686.07 ± 61.82	199.23 ± 2.76	5.96 ± .27	25.53 ± 0.55	26.4 ± 0.21	77.8	64.1	63.1	22.2	35.9	36.9

*particle concentration too dilute to measure.

Dynamic light scattering confirmed the formation of nanoscale polyplexes with hydrodynamic diameters of 120.77 ± 1.80 nm to 280.8 ± 6.70 nm (**Figure 1D** **)**. Following complete complexation of polyplexes (2:1 ratio), polyplex size increased with increasing Trp2R_9_:CpG ratio. Size measurements could not be obtained below 1:2 Trp2R_9_:CpG ratios, due to the low concentration of particles at this assembly ratio. Surface potential measurements revealed that at lower Trp2R_9_:CpG ratios (i.e. 1:1), polyplexes exhibit negative surface charges, while at higher N:P ratios, the surface potential increased with the relative amount of Trp2R_9_ in the polyplexes (**Figure 1E** **)**. Charges ranged from -34.43 ± 0.69 mV to 25.4 ± .50mV, demonstrating the tunability of surface charge. As expected, reversal of zeta potential from negative to positive was found to occur between 1:1 and 2:1 Trp2R_9_:CpG, corresponding to the ratio at which the positive and negative charges between Trp2R_9_ and CpG are approximately balanced (i.e., a passing through a charge ratio of 1).

### CpG Is Protected From Enzyme Degradation When Complexed With Trp2R_9_

Nucleic acid TLR ligands delivered *in vivo* are exposed to nucleases in the extracellular environment or within endosomes that may degrade nucleic acids and inhibit TLR activation. Improving stability may not only increase half-life allowing for better bioavailability, but prolong adjuvant persistence which is important in the generation of more potent and longer-lasting immune responses. Thus, we next tested if condensation of Trp2R_9_/CpG polyplexes could protect CpG from enzymatic degradation. Cy5-labelled CpG in soluble form or complexed with Trp2R_9_ were incubated with DNAse and the fluorescent intensity was measured to quantify the amount of DNA present before and after degradation. These studies revealed that polyplexes significantly reduced CpG degradation across all charge ratios relative to free CpG, even at lower Trp2R_9_:CpG ratios that only poorly or weakly condensed CpG (**Figure S1** **)**. Thus, these wholly immunological polyplexes also maintain the cargo-protecting ability associated with conventional polyplexes that require synthetic polymers to condense cargo.

### Polyplexes Colocalize Delivery of CpG and Trp2 and Increase Antigen Uptake by DCs

**Figure 2:**
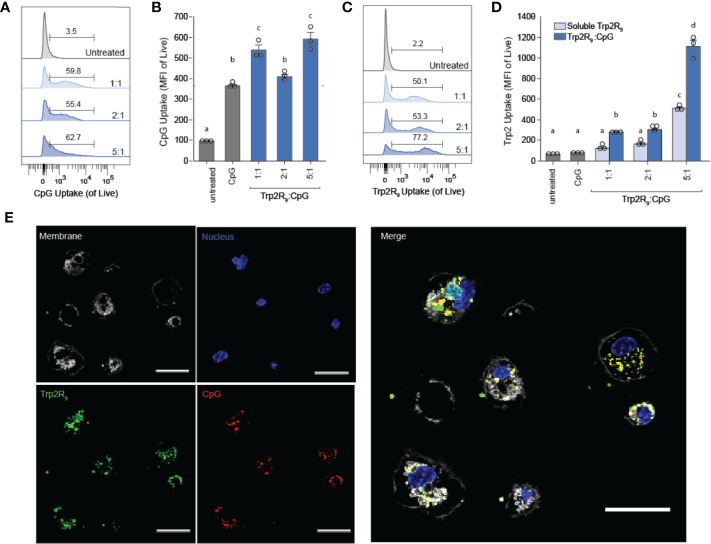
Trp2R_9_/CpG polyplexes improve immune signal uptake by DCs. **(A)** Representative gates and **(B)** quantification of CpG uptake by DCs following 24h incubation as measured by flow cytometry using Cy5-labelled CpG. **(C)** Representative gates and **(D)** quantification of Trp2R_9_ uptake was measured using FITC-Trp2R_9._ Quantitative analysis was performed to compare CpG uptake with soluble CpG controls or dose-matched Trp2R_9_. **(E)** Confocal images of CpG and Trp2 uptake in DCs (scale bar = 30μm) demonstrate colocalization of immune signals within treated DCs. Different letters indicate statistical significance among means (p <.05).

Because CpG is an agonist of TLR9, which is expressed intracellularly in endosomes, polyplexes must be internalized by DCs to initiate immunostimulatory signaling cascades. We hypothesized that the particulate nature of polyplexes would improve DC uptake compared to soluble controls. To test this, we next studied the immunological processing of complexes by measuring the level at which polyplexes were internalized by primary DCs. DCs were isolated from mouse spleens and incubated with polyplexes formed from fluorescently-labelled CpG and Trp2R_9_ for 24 h. Corresponding dose-matched free CpG and Trp2R_9_ were also included as controls. Flow cytometry analysis revealed that polyplexes generally provided a significant increase in both CpG (**Figures 2A, B** **)** and antigen (**Figures 2C, D** **)** relative to soluble forms of CpG and Trp2R9, respectively. This was indicated by increases in the mean fluorescence intensities of each signal. Antigen uptake was dose-dependent with the greatest Trp2R_9_ uptake associated with the 5:1 Trp2R_9_:CpG ratio; this ratio also contained the highest loading of Trp2R_9_.

Initiation of immune responses requires simultaneous presentation of antigen and costimulatory signal. To assess whether polyplexes conferred co-delivery of immune signals within cells, DCs were also plated onto glass-bottom dishes, treated with polyplexes, and imaged by confocal microscopy. Internalized Trp2R9 and CpG signal was localized to similar regions within treated DCs, indicating intra-cellular co-localization of the cargo (**Figure 2E**). This ability to co-delivery signals is important to promote efficient adaptive immune responses, which require encounter of both antigen (e.g.Trp2) and stimulatory (e.g. CpG) signal. While flow cytometry provides quantitative data, these confocal microscopy studies provide spatial information. Thus, together, the data confirm polyplexes were taken up by DCs rather and the signals were co-localized, rather than a more general association with the extracellular surface of DCs.

### Trp2R_9_/CpG Polyplexes Activate DCs

**Figure 3:**
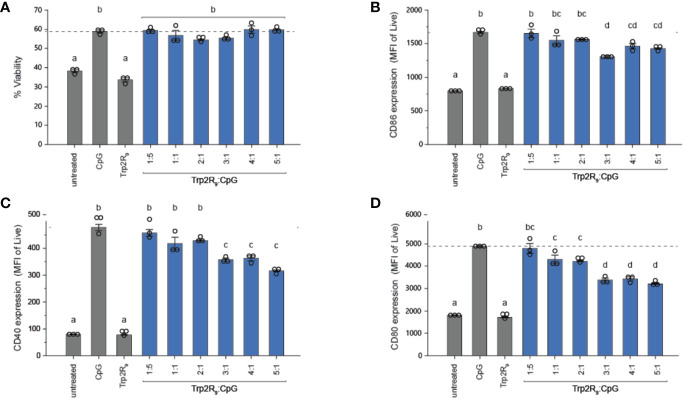
Trp2R_9_/CpG polyplexes are non-toxic and maintain ability to activate DCs. **(A)** Viability of treated DCs was measured by quantifying DAPI^-^ cells. DC activation was measured by staining with fluorescent antibody conjugates for immunostimulatory markers **(B)** CD86, **(C)** CD40, and **(D)** CD80 and analyzed for expression levels by flow cytometry. Different letters indicate statistical significance among means (p <.05).

To determine how polyplex uptake impacts DC activation, DCs were next treated with polyplexes ranging from 1:5 to 5:1 ratios. In these studies, polyplexes did not impact viability relative to other CpG-activated cells **(** **Figure 3A**). Compared to untreated cells and cells treated with soluble Trp2R_9_, Trp2R_9_/CpG polyplexes significantly increased expression of classical surface activation markers CD40, CD86, and CD80 (**Figures 3B–D**). Interestingly, at higher Trp2R_9_:CpG ratios – which exhibited the most positive potentials (**Figure 1C**) and likely the strongest binding affinity between components, the polyplexes displayed reduced DC activation compared to soluble CpG; this was despite a constant fixed CpG dose across samples. In control studies, polyplexes formed with a non-immunostimulatory oligonucleotide in place of CpG did not cause any activation, suggesting that any activation activity observed with polyplexes is driven by the CpG component condensed in the polyplexes (**Figure S2**). Together, these results indicate that while the presence of CpG confers polyplex immunogenicity, the affinity and degree of condensation as a result of Trp2R_9_ binding may influence the availability of CpG to stimulate TLR9.

### Polyplexes Activate Trp2-Specific CD8^+^ T Cells

**Figure 4:**
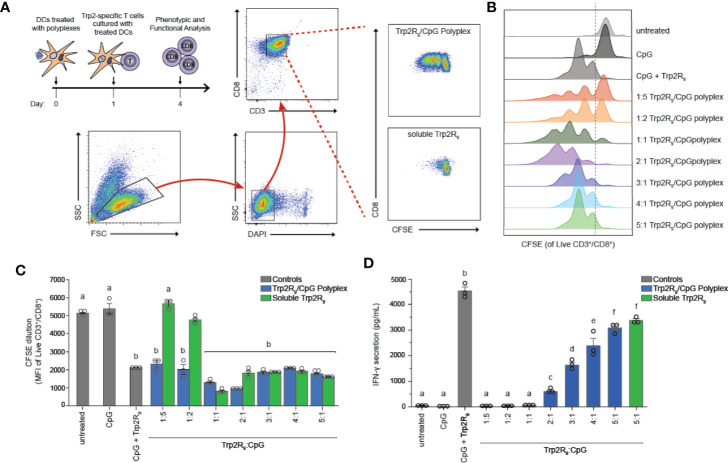
Trp2R_9_/CpG polyplexes promote antigen-specific T cell proliferation. DCs were treated with a library of Trp2R_9_/CpG polyplex ratios, soluble CpG, or soluble Trp2R_9_. After 24 h, T cells labeled with CFSE—a proliferation dye diluted with each generation of cell division—was added to culture. **(A)** Schematic of experimental set-up and representative gating schemes for flow cytometry analysis. Comparison is for proliferation of 1:5 Trp2R_9_:CpG polyplexes and dose matched soluble Trp2 **(B)** Flow cytometry histograms for CFSE dilution illustrating distinct generations of T cell proliferation different proliferation profiles for each tested ratio of Trp2R_9_:CpG **(C)** MFI of CFSE of CD3^+^/CD8^+^ cells following 72 h of DC/T-cell co-culture revealed that all complexes expanded Trp2-specific T cells with lower Trp2R_9_:CpG ratios displaying increased levels of proliferation compared to dose-matched soluble Trp2 alone. **(D)** Higher ratios of Trp2R_9_:CpG display increased levels of IFN-γ levels secreted in supernatants of DC/T cell co-cultures as measured by ELISA. Different letters indicate statistical significance among means (p <.05).

The above results indicate that polyplexes differentially alter DC activation, a step that is critical in initiating and potentiating antigen-specific T cell responses. Antigen dose, however, can also play a critical role in shaping the magnitude and nature of adaptive immunity responses to cancer or infection. Thus, we next tested if different Trp2R_9_:CpG ratios alter T the functional response of Trp2-specific T cells. In these experiments, splenic CD11c^+^ DCs were isolated from mice and treated with polyplexes and cultured with CD8^+^ T cells isolated from Trp2 transgenic mice (**Figure 4A** **)**. CD8^+^ T cells from these mice display T cell receptors specific for Trp2, and thus expand and secrete cytokines upon encountering antigen presentation with the appropriate co-stimulatory signals. Isolated T cells were labelled with fluorescent proliferation dye that becomes diluted with each generation of proliferation. Following co-culture for 72h, CD8^+^ T cells were analyzed by flow cytometry to quantify proliferation. Interestingly, altering Trp2R_9_:CpG ratios resulted in markedly different proliferation profiles (**Figure 4B** **)**. Notably, lower ratios of Trp2R_9_:CpG resulted in a heterogeneous population of T cells comprised of proliferated and unproliferated T cells that had undergone different levels of cell division, as indicated by the presence of several peaks across different intensities. Conversely, higher ratios of Trp2R_9_:CpG resulted in a more uniform population of T cells undergoing similar levels of cell division, displaying only a few peaks over a narrow range of fluorescent intensity. While T cells treated with soluble CpG did not proliferate, all polyplex formulations resulted in T cells that strongly proliferated at similar levels to soluble CpG + Trp2 treated wells, as quantified by decreasing signal intensity of the proliferation dye (**Figure 4C** **)**. This confirms the antigen-specific nature of this response. Further, at lower Trp2R_9_:CpG ratios, combined delivery of CpG with Trp2R_9_ in polyplexes significantly improved T cell expansion compared to soluble Trp2R_9_ alone. However, these findings were not observed in polyplexes with higher Trp2R_9_:CpG ratios, which expanded T cells at similar levels to soluble Trp2R_9_. Notably, due to fixed CpG levels, lower Trp2R_9_:CpG ratios contain less Trp2R_9_, suggesting co-delivery with CpG may be of particular importance at lower antigen doses to drive strong antigen-specific T cell proliferation.

To test if Trp2R_9_:CpG polyplexes also altered T cell function, we measured interferon-gamma (IFN-γ) levels from culture supernatants by ELISA. IFN-γ is a key inflammatory cytokine and important for augmenting CD8^+^ T cell cytotoxic function for enhancing anti-tumor and anti-viral effects (33). In these studies, IFN-γ secretion increased with increasing Trp2R_9_:CpG ratio; the 5:1 Trp2R_9_:CpG polyplexes displayed significantly higher levels of IFN-γ levels compared to all other tested ratios and similar levels to dose-matched soluble Trp2R_9_ control (**Figure 4D** **)**.

### Polyplex Size, Charge, and Antigen Loading Can Be Tuned

**Figure 5:**
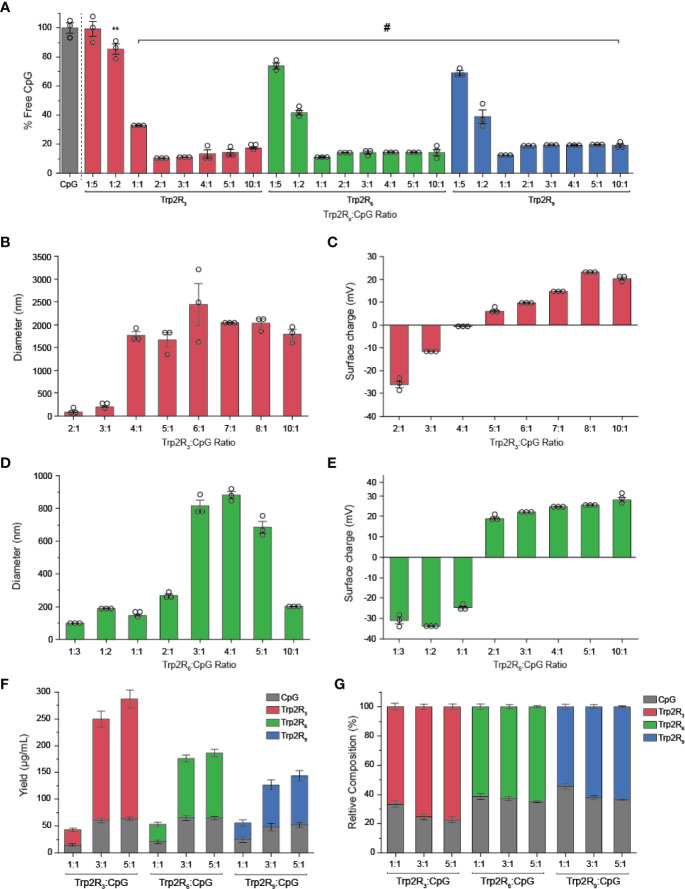
Trp2/CpG polyplexes can be assembled with peptide modified with different numbers of arginine groups to form a diverse set of polyplexes with distinct size, charge, and loading characteristics. **(A)** SYBR Green exclusion assay demonstrate formation of polyplexes using Trp2 modified with R_3_, R_6_, and R_9_. The hydrodynamic diameter **(B, D)** and **(C, E)** surface charge varied across Trp2R_x_ : CpG ratio for complexes formed with Trp2R_3_ and Trp2R_6_. Immune signal composition of assembled polyplexes was examined by analyzing **(F)** absolute and **(G)** relative loading. All significant comparisons are vs. soluble CpG and indicated, **p < 0.01, ^#^p < 0.0001.

The above results suggest that antigen dose plays an important role in T cell functionality. Thus, to further tune the dose of antigen and the relative number of epitopes – the number of copies of Trp2 delivered – we next used CpG to condense a series of alternate antigen designs in which Trp2 was modified with fewer arginine residues (i.e. Trp2R_3_, Trp2R_6_); this effectively increases the number of epitope copies at a fixed antigen dose. Using a fixed mass of CpG, polyplexes were formed by varying the mass ratio of Trp2R_x_ : CpG from 1:5-10:1. SYBR green exclusion assays revealed that Trp2R_3_ and Trp2R_6_ condensed CpG above 1:1 Trp2R_x_ : CpG ratios, as indicated by a significant reduction in fluorescence (**Figure 5A** **)**. Dynamic light scattering revealed that condensation of CpG with Trp2R_3_ resulted in smaller particles at 2:1 and 3:1 ratios (< 200nm), while polyplexes above 4:1 Trp2R_3_:CpG ratio were much larger in size with particles ranging from 1664.33 ± 160.7 nm to 1791.03 ± 102.70 nm (**Figure 5B** **)**. Sizes for ratios below 2:1 Trp2R_3_:CpG could not be measured due to the low concentration of particles forming at this range or ratios. Surface charge ranged from -24.57 ± 0.21 mV to 25.53 ± 0.55 mV. As with Trp2R_9_ complexes, surface charge increased with increasing Trp2R_3_:CpG, however, charge inversion from negative to positive occurred at a higher Trp2R_3_:CpG ratio of 2:1 (**Figure 5C** **)**. This shift in zeta potential concurs with the ratio at which the Trp2R_3_:CpG charge ratio is neutral (**Table 1**). Complexes formed using Trp2R_6_ resulted in polyplexes that varied over a greater range of sizes compared to polyplexes formed using Trp2R_9_, with hydrodynamic diameters of 145.83 ± 6.87 nm to 882.40 ± 37.08 nm (**Figure 5D** **)**. Zeta potential measurements revealed that the surface charge for Trp2R_6_/CpG polyplexes ranged from -24.57 ± 1.21 mV to 28.03 ± 1.93 mV (**Figure 5E** **)**. Ratios below 1:1 exhibited negative surface charges, while at higher ratios, polyplexes were increasingly positively charged as a function of Trp2R_6_:CpG ratios and as predicted by changes in charge ratio.

We next measured immune signal loading into complexes to assess how antigen composition was altered by using different levels of arginine modification and altering Trp2R_x_ : CpG ratios. In these studies, polyplexes formed using Trp2 with fewer arginine modifications (i.e. Trp2R_3_) resulted in higher absolute yields of antigen within complexes (**Figure 5F** **)**. 1:1 Trp2R_x_ : CpG ratios resulted in much lower yields of complexed immune signals. Similarly, the relative Trp2R_x_ and CpG composition of polyplexes could be varied across different levels of arginine modifications and changes in Trp2R_x_ : CpG ratio, with 5:1 Trp2R_3_:CpG polyplexes displaying the highest levels of antigen loading (**Figure 5G** **)**. Comparisons of the polyplex diameter, charge, and loading between polyplexes formed by condensing CpG with Trp2R_3_, Trp2R_6,_ and Trp2R_9_ are provided in **Table 1**.

### Polyplexes With Greater Amounts of Arginine Residues Drive Increased Antigen Uptake by DCs

**Figure 6:**
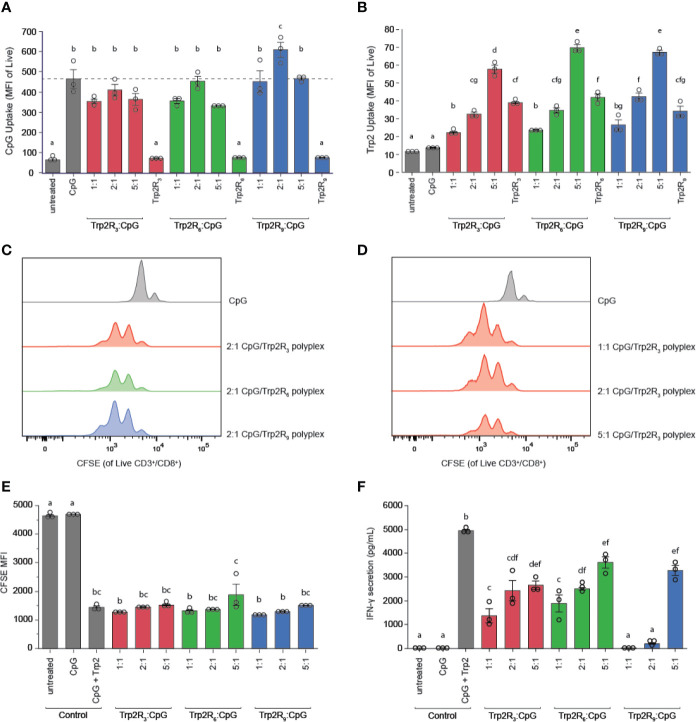
Polyplexes with different arginine modifications on Trp2 retain ability to deliver immune signals and expand T cells, but Trp2 loading in polyplexes influences T cell functionality. Flow cytometry was used to measure **(A)** CpG uptake and **(B)** Trp2R_x_ uptake following 24h incubation with Trp2R_3_, Trp2R_6_, or Trp2R_9_ polyplexes. Representative histograms for T cells cultured with treated DCs demonstrate similar levels of T cell proliferation across different **(C)** numbers of arginine modification and **(D)** Trp2R_x_ : CpG ratio, with **(E)** quantification of proliferation by dilution of CFSE MFI. **(F)** ELISAs of supernatants from DC/T cell co-cultures reveal that IFN-γ secretion levels vary with number of arginine modifications and Trp2R_x_ : CpG ratio, with higher IFN-γ correlating with formulations with higher Trp2 loading. Different letters indicate statistical significance among means (p <.05).

The physicochemical properties of immune signal carriers play an important role in altering immune responses. For example, positive surface charges can improve carrier interactions with negatively charged cell membranes, and several studies demonstrate nanoparticles may allow for more efficient uptake compared to larger micron-sized particles. Due to the differences in size, charge and loading of polyplexes, we next compared polyplex uptake by DCs across varying ratios and number of arginine residues. In these studies, DC uptake of CpG was similar to soluble CpG across nearly all polyplexes tested, with the exception of 2:1 Trp2R_9,_ which showed increased CpG uptake (**Figure 6A**). In line with these findings, DC activation studies revealed that all polyplexes activated DCs on similar levels compared to soluble CpG controls (**Figure S3**). Notably, however, Trp2R_9_ complexes displayed slightly lower levels of activation markers across CD86, CD40, and CD80. These findings may be attributed to tighter condensation of Trp2R_9_ binding, which may influence the availability of CpG and Trp2 for DC activation and antigen presentation to T cells, respectively. To evaluate Trp2 uptake, soluble Trp2R_3_, Trp2R_6_, and Trp2R_9_ were dose-matched to the highest level of Trp2R_x_ given for each set of arginine modification (i.e. 5:1 Trp2R_x_ : CpG) (**Figure 6B**). At this dose of antigen, polyplexes increased Trp2R_x_ uptake compared to their soluble Trp2R_x_ counterparts. In this study, the most pronounced differences in increased antigen uptake was observed with increasing TrpR_x_ : CpG ratio. Trp2R_x_ uptake, however, only modestly increased with increasing number of arginine residues (i.e Trp2R_3_ vs. Trp2R_6_ vs. Trp2R_9_).

### Polyplexes With Different Arginine Modifications Retain Ability to Expand T Cells, With Higher Trp2 Loading Displaying Improved T Cell Functionality

Because polyplexes formed with different arginine modifications displayed different antigen compositions, we next tested if different levels of arginine modifications altered T cell proliferation and function. In these studies, TrpR_x_ : CpG polyplexes displayed similar levels of proliferation across both number of arginine modifications and TrpR_x_ : CpG ratio (**Figures 6C–E**). However, although CpG levels were fixed across ratios, we observed marked differences in IFN-γ levels, which increased with increasing Trp2R_x_ : CpG ratio (**Figure 6F**). Polyplexes formed using Trp2 modified with R_3_ and R_6_, which have improved Trp2 loading over Trp2R_9_/CpG polyplexes, also promoted more IFN-γ secretion suggesting that Trp2 loading in polyplexes plays a role in promoting T cell functionality.

### Trp2-CpG Polyplexes Slow Tumor Growth and Increase Survival Time in Mice

**Figure 7:**
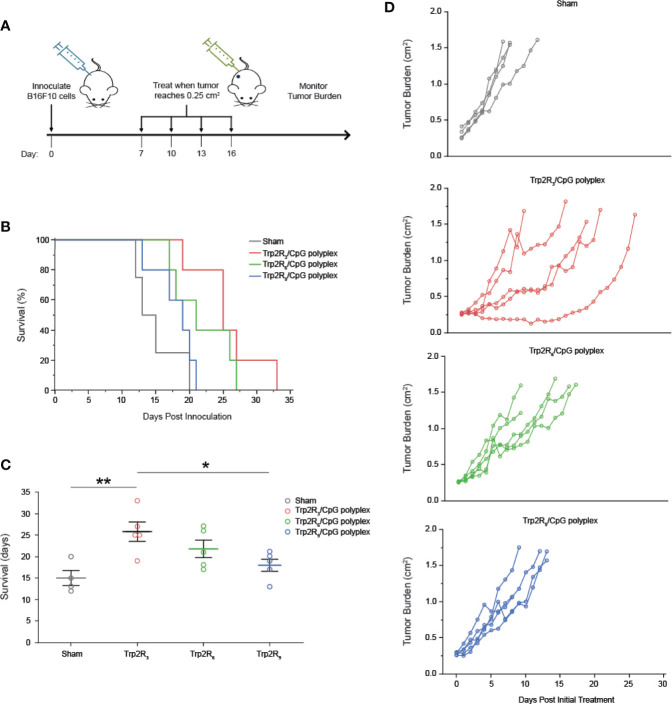
Trp2/CpG polyplexes slow tumor growth and increase survival time in mouse melanoma model. **(A)** Schematic of treatment regimen. Mice were inoculated with tumors and treated with either: 1) PBS (Sham) and polyplexes comprised of CpG condense with Trp2 modified with 2) R_3_ 3) R_6_ 4) R_9_. Mice were treated every 3 days for 4 treatments total. **(B)** Survival curves and **(C)** mean survival for each treatment. All significant comparisons are indicated *p < 0.05, **p < 0.01. Survival was measured as days post innoculation. **(D)** Tumor burden curves for each individual mouse in the study. All significant comparisons are indicated *p < 0.05, **p < 0.01.

Having identified a link between antigen dose and activation of adaptive immune responses, we next tested if the relative amount of antigen in polyplexes impacted tumor progression in a mouse model of melanoma. For these studies, we leveraged different levels of arginine modifications to alter the number of epitopes delivered. Mice were inoculated with 3 × 10^5^ B16-F10 cells in the right hind flank (**Figure 7A** **)**. When tumors reached an aggregate tumor size of 0.25 cm^2^ (~ 7 days following inoculation), mice were treated *s.c.* at the tail base on the tumor draining side with either PBS (sham) or polyplexes formed from Trp2R_3_, Trp2R_6_, or Trp2R_9_ at 3:1 Trp2R_x_ : CpG ratio. Mice received three additional treatments at 3 day intervals. In these studies, the dose of CpG was constant in all groups containing oligonucleotide. Mice treated with sham exhibited mean survivals of 15 days, while polyplexes formed from Trp2R_3_ significantly improved survival to 25.8 days (**Figures 7B, C** **)**. Polyplexes formed from Trp2R_6_ and Trp2R_9_ also showed a modest improvement in survival of 21.8 days and 18 days, however, these were not significant. The improvements in survival were also reflected in the relative rate of increase in tumor burden when assessing individual animals in each group (**Figure 7D**). In agreement with *in vitro* results, this study demonstrates that the ability of polyplexes to tune antigen dose and loading can be leveraged to promote significant improved outcomes during cancer vaccination.

## Discussion

Biomaterials offer many opportunities to improve upon current cancer therapies, many of which lack efficacy and fail to prevent relapse. We approached this challenge by harnessing the unique features of polyplexes to enable co-delivery of self-assembled melanoma antigen (Trp2) and a TLR agonist (CpG) in the absence of other carrier components. This approach can facilitate delivery of immune signals at high density to improve activation of potent immune responses. Designing simple, “carrier-free” vaccines provides an opportunity to develop insight into how each immune component impacts vaccine response to inform rational design of future vaccines. Additionally, we demonstrate that the modularity of this platform allows for incorporation of different antigen analogues to alter relative epitope concentration and physicochemical properties such as binding affinity, charge, and size. Importantly, we show that the underlying assembly processes are not impact by changes in the number of arginine modification, which is necessary to alter the amount of antigen in polyplexes. By assembling polyplexes with Trp2 and CpG, Trp2 was more efficiently internalized by DCs, colocalizing with CpG within cells. By tuning epitope concentration, we show that higher levels of antigen loading in polyplexes can promote improved antigen-specific T cell functionality *in vitro*, which translate to improved antitumor responses *in vivo*.

Polyplexes offer the opportunity to eliminate the presence of extraneous carrier materials in cancer vaccines while maintaining the features of traditional polymer platforms to leverage some of the physicochemical properties of biomaterial-based strategies. As one example, we found that polyplexes promoted improve antigen uptake compared to soluble antigen, which may be attributed to the particulate nature of complexes facilitating endocytosis. Additionally, modifying Trp2 with arginine residues results in a polyR tail, a motif commonly found in cell-penetrating peptides, which may drive transcytosis. This could be an interesting future direction to decipher the specific combination of internalization mechanisms, potentially as another level for controlling antigen and immune signal processing for self-assembled materials. Tunable surface charge can also influence uptake as cationic particles can improve material interactions with the negatively charged cell membranes. Surprisingly, in our studies, condensation of negatively charged CpG to form positively charged particles did not improve uptake compared to soluble CpG. One possibility is that surface charge may alter the mechanism of cellular uptake and subsequent intracellular trafficking, which can hinder uptake. Binding affinity may also affect the dissociation and subsequently the delivery and processing of immune signals within cells (34, 35).

Leveraging polyplex principles that require internalization in endosomes followed by escape into the cytosol for transcription and translation, our data demonstrates that polyplexes enable two distinct yet critical delivery routes. First, the ability of complexes to activate DCs suggests that polyplexes allow for the delivery and binding of CpG to receptors in endosomes to activate TLR pathways. Secondly, antigen delivered within cells can undergo endosomal escape into the cytosol for loading onto MHC-I, a critical step for activating CD8^+^ T cells. Elucidating the precise trafficking mechanisms that facilitate polyplex processing through these pathways can offer valuable insight towards improving co-delivery of immune components.

We also observed some interesting differences in how CpG behaved in complexes versus soluble form. During DC activation studies with Trp2R_9_ polyplexes, although Trp2R_9_:CpG promoted high expression of activation markers, polyplex treated DCs were less activated compared to free CpG alone, and activation was further attenuated with increasing Trp2R_9_:CpG (**Figures 3B–D**). One potential explanation is the strong binding between Trp2R_9_ and CpG at higher ratios of Trp2R_9_:CpG which could require a longer time for binding and processing or limit the availability of CpG to TLR9 receptors. Although the SYBR Green exclusion assay suggested similar binding levels (**Figure 1B**), more sensitive measurements of binding affinity such as surface plasmon resonance might provide additional insight into some of the different effects. Despite the less efficient activation of DCs by some Trp2R_x_ : CpG ratios, all polyplex formulations still promoted strong levels of activation and further, provide other benefits such as improved antigen uptake and protection from enzyme. Importantly in our studies, treatment of cells with free Trp2R_x_ or Trp2R_x_-ODN polyplexes generally did not activating cells, suggesting that CpG is the key driver of these observed effects.

Interestingly, polyplexes across all arginine modifications displayed similar levels of uptake, DC activation, and T cell expansion, despite changes in physicochemical properties for each library of Trp2 analogues. We instead observed that polyplexes formed from CpG and Trp2 modified with R_3_ generally stronger T cell responses compared to complexes modified with R_6_ or R_9._ One possibility is that Trp2R_3_ more closely resembles native Trp2 because it contains the fewest modifications and closest protein length, making it more easily processed by DCs and more readily identifiable by CD8^+^ T cells specific to Trp2. Alternatively, at a given Trp2R_x_ : CpG ratio, Trp2R_3_ complexes contained higher levels of Trp2, which may improve tumor-specific T cell activation. Many antigens expressed on tumors are also expressed on normal tissues; thus T cells specific to many tumor antigens are subject to thymic selection which deletes high avidity T-cells recognizing self-antigen (36). This leaves a repertoire of low avidity T cells that require high doses of antigen to become stimulated (37). This is corroborated by our findings that increasing the antigen concentration by altering Trp2:CpG by was found to correlate with improved T cell functionality.

Our approach reveals several important questions for future studies. First, we prepared polyplexes using different analogues of the same tumor epitope. While T cells recognize specific epitopes, tumor antigens are larger proteins which present multiple peptide fragments, each with varying degrees of immunogenicity (38). Thus, additional studies are needed to investigate if the modularity of polyplexes can be extended to other tumor epitopes. Additionally, the polyplexes prepared in these studies contain a single antigen epitope. Recent studies, however, suggest that immunotherapies that target multiple epitopes allows for improved anti-tumor responses (39, 40). As one example, EMD640744, a multi-epitope vaccine for patients with advanced solid tumors, is currently undergoing phase I clinical trials (41). Targeting multiple epitopes allows for a wider spectrum of antigens that expanded T cells can recognize within heterogenous tumor cell populations. Further, potent anti-tumor responses arise from simultaneous induction of both humoral and cellular responses, requiring different epitopes to activate B cells and multiple subsets of T-cells (42–44). Thus, there is also motivation to test if multiple epitopes can be incorporated into polyplexes. Lastly, the finding that Trp2R_3_ elicited the strongest functional T cell responses and subsequently improved tumor-burden raises some interesting implications surrounding the role of antigen in promoting anti-tumor immunity. Our results demonstrate that polyplex structures comprised of immune signals offer a tunable platform to co-deliver CpG and Trp2 peptide, while eliminating carrier components. As highlighted in our studies utilizing different Trp2 peptide modifications, the simple approach and modularity of this platform offers a unique opportunity to study the role of antigen dosing, structure, and co-delivery in stimulating effector T cell responses – this insight can contribute to new therapies to improve tumor-specific immune responses.

## Data Availability Statement

The datasets generated for this study are available on request to the corresponding author. Further inquiries can be directed to the corresponding author.

## Ethics Statement

All studies involving animals were carried out in compliance with federal, state, and local laws and followed institutional guidelines, including the Guide for the Care and Use of Laboratory Animals and the Animal Welfare Act. All experiments were reviewed and approved by the University of Maryland’s Institutional Animal Care and Use Committee (IACUC). 

## Author Contributions

CJ, ST, and AA conceived the studies. ST and AA conducted the experiments and data analysis. All authors planned and wrote the manuscript. All authors contributed to the article and approved the submitted version.

## Funding

This work was supported by in part by NIH # R01 EB027143 and United States Department of Veterans Affairs # I01 BX003690.

## Conflict of Interest

CJ is an employee of the VA Maryland Health Care System. The views reported in this paper do not reflect the views of the Department of Veterans Affairs or the United States Government. CJ has an equity position in Cellth Systems, LLC and Avidea Technologies.

The remaining authors declare that the research was conducted in the absence of any commercial or financial relationships that could be construed as a potential conflict of interest.
