# Supplementary material for: Altering Antigen Charge to Control Self-Assembly and Processing of Immune Signals During Cancer Vaccination
Source: Front Immunol. 2021 Jan 6;11:613830. doi: 10.3389/fimmu.2020.613830 (PMC7815530; doi:10.3389/fimmu.2020.613830)
Supplement: Supplementary file 1 [file DataSheet_1.docx]

Altering antigen charge to control self-assembly and processing of immune signals during cancer vaccination

Supplementary Information

Shannon J. Tsai^1^, Allie Amerman^1^, Christopher M. Jewell^1-5^

^1^ Fischell Department of Bioengineering, University of Maryland, College Park, MD 20742, USA

^2^ Robert E. Fischell Institute for Biomedical Devices, University of Maryland, College Park, MD 20742, USA

^3^ United States Department of Veterans Affairs, VA Maryland Health Care System, Baltimore, MD 21201, USA

^4^ Department of Microbiology and Immunology, University of Maryland Medical School, Baltimore, MD 21201, USA

^5^ Marlene and Stewart Greenebaum Cancer Center, Baltimore, MD 21201, USA

*** Correspondence:**Christopher M Jewell
cmjewell@umd.edu

Keywords: immunotherapy, vaccine, cancer, immune, polyplex, nanoparticle, co-delivery, self-assembly

**SUPPLEMENTARY FIGURES**

**
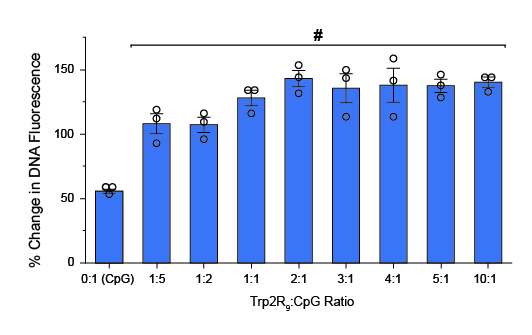
**

**Figure S1.** Trp2R_9_/CpG polyplexes protect CpG against enzymatic degradation. #p < 0.0001. Statistical comparisons are vs. 0:1 (CpG)

**
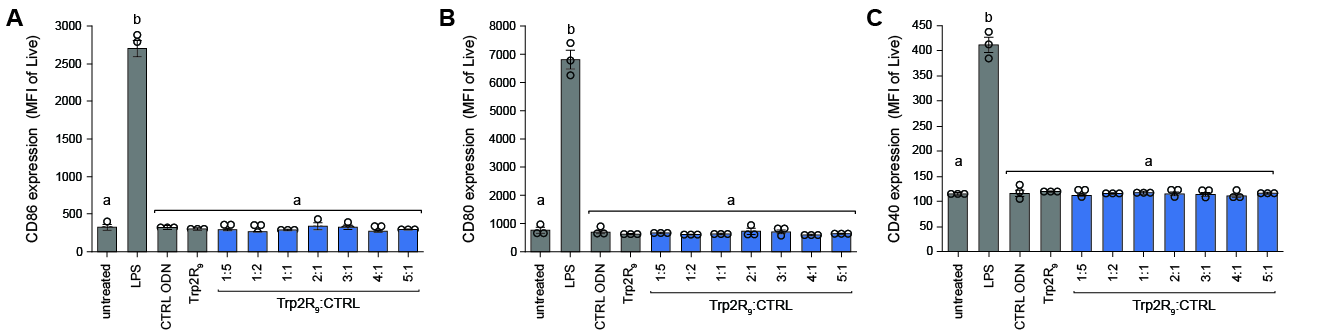
**

**Figure S2.** Trp2R_9_/CTRL ODN polyplexes do not activate DCs. Polyplexes were formed by condensing CTRL ODN in place of CpG. Flow cytometry reveal that treated DCs maintained low levels of expression for immunostimulatory markers **(A)** CD86, **(B)** CD40, and **(C)** CD80. Different letters indicate statistical significance among means (p < .05)

**
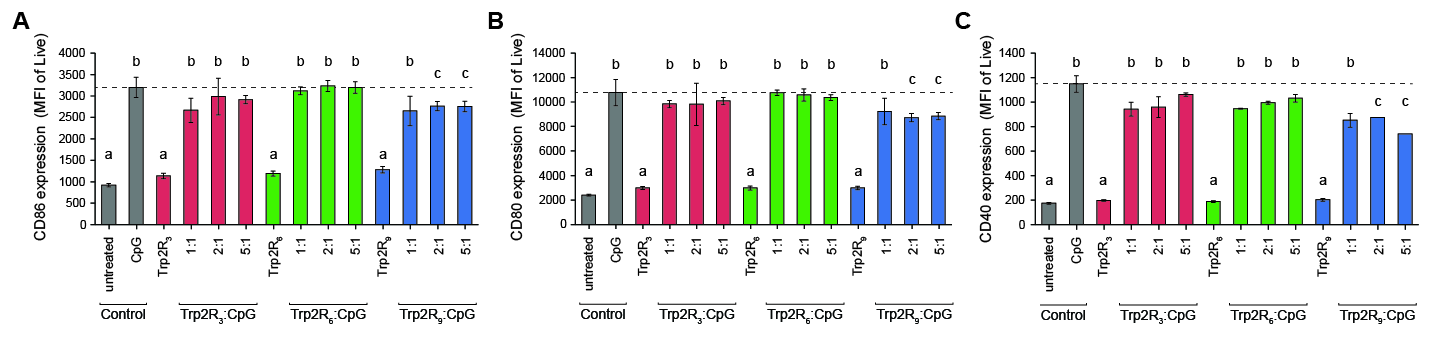
**

**Figure S3.** Polyplexes formed from Trp2 with different arginine modifications activated DCs on similar levels compared to soluble CpG controls. Different letters indicate statistical significance among means (p < .05).
